# Stray Dog Population in a City of Southern Mexico and Its Impact on the Contamination of Public Areas

**DOI:** 10.1155/2018/2381583

**Published:** 2018-09-25

**Authors:** Gloria R. Cortez-Aguirre, Matilde Jiménez-Coello, Eduardo Gutiérrez-Blanco, Antonio Ortega-Pacheco

**Affiliations:** ^1^Department of Animal Health and Preventive Medicine, Faculty of Veterinary Medicine and Animal Sciences, Autonomous University of Yucatan, AP 4-116, Merida, Yucatan, Mexico; ^2^Wau Veterinary Centre, 24040 San Francisco de Campeche, Campeche, Mexico; ^3^Regional Research Center “Dr. Hideyo Noguchi”, Autonomous University of Yucatan, CP 97000, Merida, Yucatan, Mexico

## Abstract

To assess the risk of zoonotic pathogen transmission as function of stray dog presence and health status, a cross-sectional study was carried out in a large city of southern Mexico that lacks comprehensive strategies for the control of stray canine populations. The photographic capture-recapture method was used to estimate the density of dogs/km^2^. In the same way, dog feces from 14 public parks of the city were collected to determine the prevalence and intensity of infection with gastrointestinal parasites. The canine population was estimated between 65 and 80 thousand dogs, with a population density of 1,081 dogs/km^2^, mostly males (71.4%). A high proportion of dogs (72.3%) were found to be in good body condition score (BCS 3). The person:dog ratio was 2.3. The likelihood of being in the BCS 2 category was lower in areas with a higher density of dogs. All feces collected from the parks contained eggs of intestinal parasites, most of them with a medium (42.9%) to high (35.7%) infection intensity, notably* Ancylostoma caninum*. It was recorded that cases with a low-intensity of GI infection showed polyparasitism (35.7%) associated with* A. caninum.* There is a large population of stray dogs that roam freely in the streets of Campeche city with access to sources of food, which is reflected by their good BCS, and dogs do not have access to preventive medicine programs (de-worming) and thus contaminate public parks with feces with significant parasitic egg loads of zoonotic importance.

## 1. Introduction

Dogs are very popular pets in all urban areas around the world, and their relation to humans has always been very close. In Mexico, a population of 23 million dogs has been estimated, with 70% of them classified as street dogs or stray dogs [1]. Surplus of dogs are reported in several states of Mexico [2–4], including the Yucatan peninsula where stray dogs, also known as “malix” in the region, are also abundant as a result of abandonment from their original owners [5]. Dog owners may allow uncontrolled breeding, a situation that has favored the growth of this population in the streets. Besides, if the carrying capacity of the environment is high dogs will have better body condition and more success for breeding. Traditionally, dog population control is done through surgical sterilization of males and females, a strategy whose effectiveness is questionable [6], although it might help to improve owner awareness to improve domestic dog quality of life. The lack of official programs to control dog populations may lead to surplus of dogs in a specific area. This problem, created by humans, very often ends in acts of cruelty towards dogs and public health problems, including attacks [7] and infectious diseases such as rabies, leptospirosis, intestinal parasites [8], Chagas disease [9], and leishmaniasis [10], among many others [11]. Abundance of stray dogs is associated with an increased risk of zoonotic diseases particularly in poor regions. Stray dogs roaming the streets may be owned and have access to public areas such as parks, gardens, green streets, and other areas, and their feces may be infection sources for humans, particularly when feces contain gastrointestinal parasites [12]. Zoonotic gastrointestinal parasites, mainly nematodes from dogs, are a common environmental health risk in public parks and recreational areas contaminated by dog feces across the globe, especially in the tropics [13]. Common parasites present in dog feces collected from public areas, including* Ancylostoma caninum* and* Toxocara canis*, are zoonotic and produce clinical symptoms such as cutaneous larva migrans and ocular larva migrans, respectively [14].

There are no data on the stray dog population in the city of Campeche, where no actions for control of stray dogs have been officially implemented. The objective of this study was to estimate the stray dog population, their health status, and its impact on the contamination of public areas as a basis for establishing effective control programs and thereby minimizing the impact on public health.

## 2. Materials and Methods

A cross-sectional study was conducted in San Francisco de Campeche, capital city of the State of Campeche during May to July 2016. San Francisco de Campeche city is located in the western part of the Yucatan Peninsula on the Gulf of Mexico (parallels 17°49′ and 20°51′ latitude north and the meridians 89°06′ and 92°27′ longitude west) [15].

**Figure 1:**
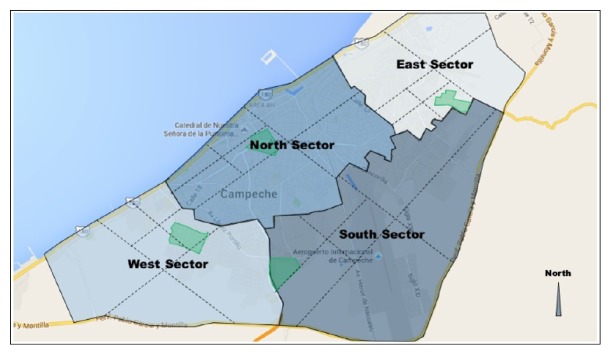
The city of San Francisco de Campeche divided into 4 sectors (North, South, East, and West); each sector divided into four from where a quadrant (in green colour) was selected and walked in foot.

The density of stray dogs was estimated using the photographic capture and recapture method [16]. This method is a modification of Schnabel's method [17]. Briefly, for convenience and trying to include all areas, the city was divided into four sectors; each sector was divided into four from where a quadrant where dogs were more prone to be detected (i.e., near markets, dumpsters) was selected and walked on foot (Figure 1). Each quadrant was walked by the same person for three days on each day from 6:30 to 08:30 and from 18:30 to 20:30. Each dog was identified individually by direct observation and photographed. Recaptures on the following day were based on the record generated previously.

The equation used was as follows:

(1) $$\begin{array}{c} \hat{K}=\frac{\sum \left(XiXm\right)}{\sum \left(Xi,m\right)+1} \end{array}$$

The formula was used to estimate the population of free roaming dogs.* Xi* is the total daily photographed dogs (captured),* Xi,m* is dogs previously captured,* K *is the estimator of the total population, and* Xm = Xi - Xi,m,* or dogs photographed for the first time and considered marked or recaptured if photographed again in subsequent days.

From the “captured” animals, information on their sex and breed was recorded. Three types of breeds were defined according to their phenotypic characteristics: (1) purebred (dogs with 100% of phenotypic characteristics of a particular breed), (2) crossbreed (dogs with mixed breed phenotype), and (3) “Malix” (mongrel) or dogs originating in the area [5].

The health status of dogs was determined by the body condition score (BCS). The scale used was from 1 to 5, with 1 being thin and 5 being obese [18]. As part of the health status, the presence of macroscopic lesions in the skin was considered. Lesions were identified as dermatological alopecic areas of pustular or flaky type, skin lesions, dermatitis, or erythema [19]. Their physiological state (apparently pregnant or lactating females) [20] and other conditions such as lameness, respiratory signs, ophthalmic discharge, and coughing were also recorded.

Samples of dog feces were collected directly from the soil of major public city parks (green areas), considered to be representative areas of influx of people and dogs and areas of logistical convenience. A total of 14 parks were selected from the four established areas for sampling. From each park 1 to 1.5 kg of feces was collected, from five equidistant points of field sampling (250-300 g per point). A homogeneous mixture was made and the amount of 100 g feces was taken for later analysis through the MacMaster technique to estimate the number of eggs per gram of feces (e/g) [21].

Quantitative and qualitative variables obtained from the study of photographed parks and dog populations were analyzed using SPSS 22.0. With the information obtained from the feces examination, prevalence, parasite morphology, and infection intensity were determined. Intensity of parasite infection was classified according to load: low (50-100 e/g), medium (150 e/g), or high (load greater than or equal to 500 e/g) [22].

Since this was an observational study with no contact with animals (feces samples collected from the soil) no ethical clearance was required.

## 3. Results

**Figure 2:**
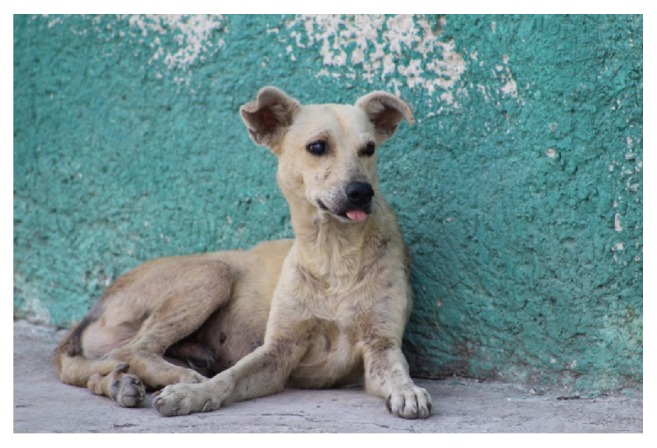
A typical “malix” free roaming male dog from San Francisco de Campeche.

Of the total number of dogs, the east sector showed the highest number followed by the south and north (Table 1). The results are expressed for the total of the four quadrants, with a lower and upper limit of 63,000 to 80,000 dogs, which represents 1,081 dogs per km^2^, and considering the currently reported population in the city (INEGI, 2010) the person/dog ratio was 2.3. Most dogs were captured during the morning shift at which time 40.1% were males and 71.4% of the total population was accounted for. The dominant type in the census population was “Malix” with 93.5% of the specimens of this type (Figure 2).

**Figure 3:**
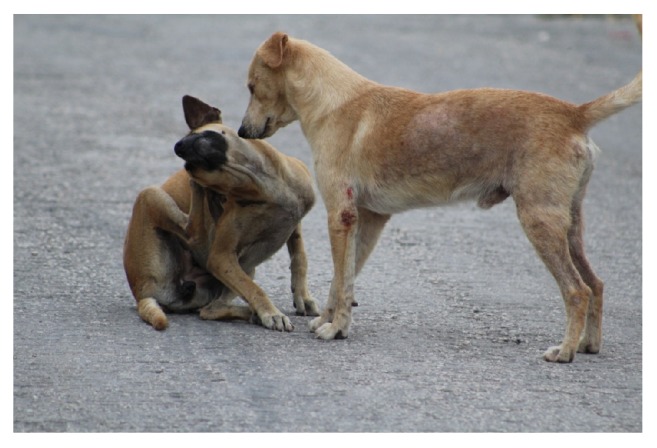
A male “malix” dog (standing) with good body condition (BCS 3) presenting a wound in the left former limb and diffuse alopecia in the thoracic region.

The most commonly BCS seen was 3, in 75.5% and 71.5% for females and males, respectively. The lower percentage of dogs with BCS 3 was seen in sector with a lower density of dogs particularly in the west of the city (Table 1). It is noteworthy that in both sexes only 0.4% had BCS 1, while no cases of BCS 5 were seen. Of the “captured” dogs, 26.0% had skin lesions (Figure 3), mostly in males (68.8%). Dogs from the southern sector of the city were most affected with skin lesions (44.8%). Of the dogs observed, 1.5% were lame and located in the western sector, where more dogs were injured; 2.3% of dogs had respiratory symptoms and were mostly found in the eastern sector. Ophthalmic discharge was common in 65.0% of the dogs, followed by coughing among 30.0% and nasal discharge among 5.0%.

All public parks studied (100%) were contaminated with dog feces, harboring parasitic eggs including* A. caninum*,* T. canis*, and* D. caninum*. In the majority of the parks (70%), dogs were observed defecating. The intensity of infection in parks by zone was medium for the eastern sector and low for the other areas of the city. The intensity of infection with type of parasitic eggs is shown in Table 2. Evaluated stool was from collected pooled samples. It is noted that eggs with the* A. caninum* parasite were most frequently found in areas with medium to high intensity of infection.

## 4. Discussion

This study generates information about the population density of stray dogs in a typical southern Mexican city lacking a comprehensive canine control program. Overpopulation of stray dogs is a reflection of the usual practice of the owners allowing their dogs to reproduce indiscriminately, coupled with the lack of public programs for dog population control. In Mexico, every year, 7,000 stray dogs are sacrificed [23]. This overpopulation is common to observe in other Latin-American countries such as Chile, where a stray dog population of 112,000 is estimated in the urban area of Santiago [24]. In Camagüey Cuba, densities reported are 45 to 75 dogs per neighborhood [25]. In the district Los Olivos in Lima, Peru, in an area of 17.3 Km^2^ 1,411 ±643 dogs were counted during the day and 922 ± 497 animals at night [26]. Similar to other developing countries, in the area of study (Campeche city), there are no strategies designed to control stray dogs; i.e., there are no animal control centers, permanent sterilization campaigns, or social awareness about responsible pet ownership. In Tezontepec Hidalgo, Mexico, for example, only 14% of males and 37% females are neutered [4], while in Brazil, the number of dogs adopted in shelters does not exceed 9.5% and 59% of the females are euthanized [27]. The dog breed that predominated in this study was Malix, similar to what was found in other cities in southern Mexico [5]. In Mexico, the Malix (or mongrel) dogs are popular among owners [2, 5] and may represent a genetic characteristic of these stray dogs.

Most of the observed dogs were moving during the morning. Beck [16] and Totton et al. [28] reported that dogs are more visible during the cooler hours of the day avoiding hot weather, particularly during the summer, when dog detection in the streets decreases [16]. The presence of dogs in the streets as seen in this study may be related to food availability, which increases when people take out the garbage to be collected by the maintenance services of the city [29]. In Mexico, a high percentage of people have animals in their yards, and most are allowed to roam without supervision. A study in Yucatan found that 63% of dogs roam without oversight and are fed with waste material, a common practice, especially in rural communities [5]. The dogs observed were mostly males. In a study, a larger population of males was found to be probably related to the lower life expectancy of the female, since neonatal and postpartum problems cause 50% of deaths [30]. People prefer male dogs because they have a more aggressive temperament to be used as guardian, and they are less tolerant of estrus in females [31]. Movement behavior was most frequently observed in males and is likely related to breeding behavior and the search for food [16].

Body condition is a crucial aspect of the health of a canine as it can influence the ability of an animal to fight against any infectious process. Good nutrition results in a good body condition, as seen in a significant portion of the animals studied. These findings are in contrast, for example, with what is described in some regions of India that have a high density of stray dogs where 49% of the population sampled was emaciated [32]. Dogs with poor BCS are less healthy and more prone to various pathogens, particularly severe helminthiasis [8]. In the present study, most dogs showed good BCS, which predicts better health over their lifetime and is more capable for reproduction and survival of offspring. It has been reported that visible cutaneous lesions can be present in up to 34% of strays, associated with scabies and others causes, in particular,* Demodex canis* (23%) [33]. In this study, the causes of skin lesions were not determined. However, a significant proportion of the dogs sampled showed lesions that were not necessarily associated with their BCS but may be associated with the high density of dogs and a high rate of transmission of mites between them. A considerable percentage (3.7%) of the dogs observed had signs of mechanical injuries and respiratory problems that may be associated with canine distemper. This viral disease is widely distributed in regions of poverty, where there are no prophylactic programs [8] and which contribute to poor animal welfare and increased mortality of the stray dogs. It is worth mentioning that the animals observed in this study have a very low probability for being immunized with vaccines other than rabies. In Taiwan, only 39.5% of the owners apply the initial vaccines and 18% of dogs have never been vaccinated [34]; rabies vaccination in dogs and cats is mandatory in Mexico and has no cost. Public parks evaluated in this study had presence of large quantities of dog stool with intestinal nematode eggs, including* T. canis* and* A. caninum*, considered the main species of gastrointestinal helminths infecting dogs worldwide. These zoonotic helminths have been found on public park grounds and in fecal samples of dogs in public parks in Europe, the Americas, Africa, and Asia [14]. In Latin-American countries, for instance, a study in Chile showed that 24.6% of the parks sampled were positive for nematode eggs and 9.23% for cestodes [35]. In the present study 100% of the parks tested positive to some parasitic zoonotic eggs; 98.2% of the parks were contaminated with nematodes and 1.4% with cestodes. In a previous study on Yucatan in dogs with free access to the street, 80% of dogs were positive for some form of gastrointestinal parasite, mostly* A. caninum*,* T. canis*, and* T. vulpis,* with high parasitic loads, particularly of* A. caninum* [36], similar to what has been observed in the present study. In the absence of deworming programs, the presence of gastrointestinal parasites in dog feces is high even with high loads of eggs [37]. In tropical conditions, embryonated eggs of the major nematodes can remain viable in dogs even for years, and the development and viability of nematodes are very successful depending on the temperature and humidity in these areas, as evidenced by a study in various localities in Costa Rica [38]. The high contamination of the parks studied in the city, as a consequence of high density of free roaming dogs in the city, has significant epidemiological relevance since there is no fecal collection system. The eggs of the different parasites found in this study can be dispersed easily with air currents, a situation that favors direct contact with or ingestion of parasites by people visiting the parks. Pooling fecal samples was used as a screening test to evaluate soil samples containing nematode eggs. Results here obtained demonstrated its feasibility and cost-effective method that gave a broad panorama of the situation. However, limitations of this strategy include low sensitivity to detect a more realistic intensity of nematode egg contamination.

## 5. Conclusions

This study found a large population of stray dogs in the city of Campeche with a high proportion being healthy and with a good body condition. The high abundance of stray dogs and lack of prophylactic programs targeting stray dogs in Campeche city generates a source of infective eggs, in particular* A. caninum*. This finding highlights a public health problem for humans cohabiting with large populations of stray dogs.

## Figures and Tables

**Table 1:** Percentage of dog numbers according to the sector of the city of San Francisco de Campeche and percentage of dogs with good body condition score (BCS 3).

Sector	Number of dogs n (%)	BCS 3 n (%)
North	17,673 (21.9)	11,664 (66.0)

South	19,206 (23.8)	15,211 (79.2)

East	32,360 (40.1)	24,173 (74.7)

West	11,459 (14.2)	7,334 (64.0)

**Table 2:** Intensity of infection with canine intestinal parasite eggs in pooled samples collected from 14 public parks in the city of Campeche.

	**Intensity of infection** **∗**		
**Parasite**	**Low**	**Medium**	**High**
***A. caninum***	14.28 %	42.9%	35.7 %

***T. canis***	14.28%	0.0%	0.0%

***D. caninum***	7.14%	0.0%	0.0%

**Total**	35.7 %	42.9 %	35.7 %

*∗*Low 50-100e/g, Medium 101-500 e/g, and High ≥500 e/g.

## Data Availability

The data used to support the findings of this study are available from the corresponding author upon request.
